# Localization of chondromodulin-I at the feto-maternal interface and its inhibitory actions on trophoblast invasion *in vitro*

**DOI:** 10.1186/1471-2121-12-34

**Published:** 2011-08-18

**Authors:** Shigenori Miura, Chisa Shukunami, Kaori Mitsui, Jun Kondo, Yuji Hiraki

**Affiliations:** 1 Department of Cellular Differentiation, Institute for Frontier Medical Sciences, Kyoto University, Kyoto 606-8507, Japan; 2 Research and Development Division, Science and Technology Research Center Inc., Mitsubishi Chemical Group, Kanagawa 227-8502, Japan; 3 Advanced Medical Research Laboratory, Research Division, Mitsubishi Tanabe Pharma Corporation, Kanagawa 227-0033, Japan

## Abstract

**Background:**

Chondromodulin-I (ChM-I) is an anti-angiogenic glycoprotein that is specifically localized at the extracellular matrix of the avascular mesenchyme including cartilage and cardiac valves. In this study, we characterized the expression pattern of ChM-I during early pregnancy in mice *in vivo *and its effect on invasion of trophoblastic cells into Matrigel *in vitro*.

**Results:**

Northern blot analysis clearly indicated that *ChM-I *transcripts were expressed in the pregnant mouse uterus at 6.5-9.5 days post coitum. *In situ *hybridization and immunohistochemistry revealed that ChM-I was localized to the mature decidua surrounding the matrix metalloproteinase-9 (MMP-9)-expressing trophoblasts. Consistent with this observation, the expression of *ChM-I *mRNA was induced in decidualizing endometrial stromal cells *in vitro*, in response to estradiol and progesterone. Recombinant human ChM-I (rhChM-I) markedly inhibited the invasion through Matrigel as well as the chemotactic migration of rat Rcho-1 trophoblast cells in a manner independent of MMP activation.

**Conclusions:**

This study demonstrates the inhibitory action of ChM-I on trophoblast migration and invasion, implying the potential role of the ChM-I expression in decidual cells for the regulated tissue remodeling and angiogenesis at feto-maternal interface.

## Background

Chondromodulin-I (ChM-I) is a naturally occurring anti-angiogenic glycoprotein that localizes to the avascular domains of mesenchymal tissues such as cartilage, cardiac valves, and the eye [1-3], where angiogenesis is strictly limited. Using both recombinant and adenovirally expressed ChM-I protein, we have demonstrated that ChM-I inhibits the migration, proliferation, and tube morphogenesis of cultured vascular endothelial cells, and suppresses tumor angiogenesis [4-8]. Moreover, ChM-I-deficient mice show a vascularized phenotype in their aged cardiac valves, which is caused by a loss of anti-angiogenic valvular functions and abnormal vascular invasion [2]. These studies suggest that ChM-I may serve as a matrix component that confers anti-angiogenic resistance to specific mesenchymal tissues.

During mouse skeletal development, expression of *ChM-I *was dramatically increased in association with cartilage formation. *In situ *hybridization revealed that the transcripts were detected at the sites of chondrogenesis such as the occipital bone rudiments at 11 days p.c. in mouse embryos. Then the specific expression of *ChM-I *became apparent in all cartilaginous skeletal elements in the body including the nasal septum, tracheal rings, ribs, and vertebral column [9]. Prior to chondrogenesis, *ChM-I *transcripts were detected in cardiac valve precursor cells of the heart at 9.5 days post coitum (p.c.) and its expression persisted in cardiac valves in the adult [2].

In the course of our study to explore the sites of *ChM-I *expression in early stages of development, we carried out northern blot analysis of pregnant mouse uterus and found it to be a prominent expression site at 7.5 day p.c. or later. Then, after detailed analysis, we unexpectedly found that intense (or strong) hybridization signals were detected in maternal tissues such as decidua rather than embryos at this early stage of pregnancy. In this study, we determined the differentiated mouse decidua as a novel site of *ChM-I *expression and found that ChM-I transcripts were induced by the decidualization of endometrial stromal cells *in vitro*. We examined here a possible involvement of ChM-I in angiogenic events and tissue remodeling of decidua by a Matrigel invasion assay *in vitro*.

## Results

### Expression of ChM-I in the decidua during the early implantation period

**Figure 1:**
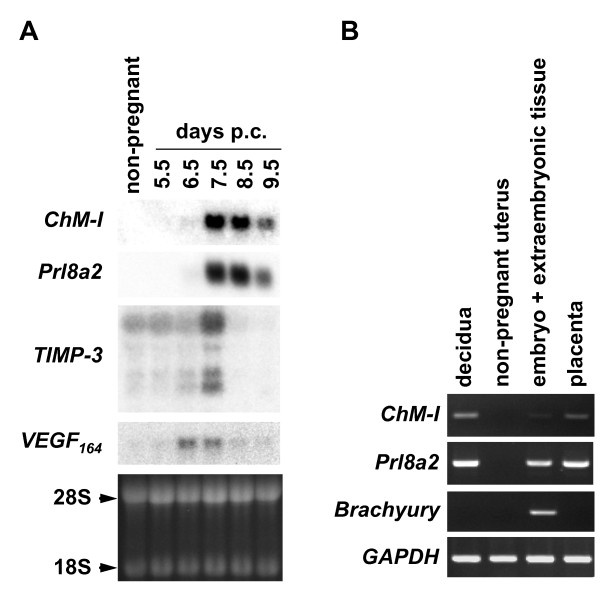
**Expression of ChM-I in the mouse uterus during early pregnancy**. (A) Northern blot analysis of *ChM-I *mRNA in the pregnant mouse uterus. Total RNA was extracted from pregnant mouse uteri (myometrium + decidua + embryo) and non-pregnant mouse uteri, separated on a denatured agarose gel, and transferred to a nylon membrane. The blots were then hybridized with a radio-labeled probe for *ChM-I, Prl8a2, TIMP-3*, and *VEGF-A_164_*, respectively. The equivalent loading of RNA (15 μg/lane) in each lane was verified by ethidium bromide staining. Arrowheads indicate the positions of the 28S and 18S ribosomal RNAs. (B) Total RNA was extracted from decidua (7.5 days p.c.), non-pregnant mouse uteri (8-week-old), embryos (7.5 days p.c., including extraembryonic tissues), and placenta (13.5 days p.c.). The decidual tissues were prepared by the removal of embryos and extraembryonic tissues from whole decidual capsules. One microgram of each total RNA preparation was reverse-transcribed, and the expression of *ChM-I *and marker genes (*Prl8a2*, a marker for trophoblasts and decidua; *Brachyury*, a marker for embryonic tissue) was analyzed by RT-PCR (30 cycles). *GAPDH *was used as an internal control. The data are representative of three independent experiments.

In mice, implantation occurs at between 4.5-5.0 days p.c. and triggers the transformation of uterine stroma into the cohesive spongy tissue called decidua. This event accompanies drastic tissue remodeling via the uterine angiogenesis and the invasion of trophoblasts to make feto-maternal connections required for the maintenance of pregnancy. By northern blot analysis, *ChM-I *transcripts were undetectable in the uterus at 5.5 days p.c. as well as in the non-pregnant mouse uterus (Figure 1A). These transcripts became barely detectable at 6.5 days p.c. and were at their most abundant levels at 7.5 days p.c. The expression levels gradually declined from the onset of placentation (around 8.5 days p.c.) onward. The temporal pattern of *ChM-I *expression was thus found to be similar to that of *Prl8a2 (prolactin family 8, superfamily a, member 2) *(Figure 1A), which is expressed in the trophoblasts and the decidua [10]. In contrast, transcripts for TIMP-3 (tissue inhibitor of matrix metalloproteinase-3) and VEGF-A_164 _(vascular endothelial growth factor-A_164_), both known angiogenesis-related gene products expressed in the decidua, increased until 7.5 days p.c., and then rapidly disappeared. As shown in Figure 1B, RT-PCR analysis of a pregnant mouse uterus (7.5 days p.c.) indicated that *ChM-I *expression was strongly detected in the maternal decidua that also expressed *Prl8a2*, but was marginal in the embryonic tissue that was positive for *Brachyury*, a marker of the mesoderm [11]. The placenta (13.5 days p.c.), which is formed through the fusion of the decidua, EPC (ectoplacental cone), chorion, and parts of the allantois, was also positive for *ChM-I*.

**Figure 2:**
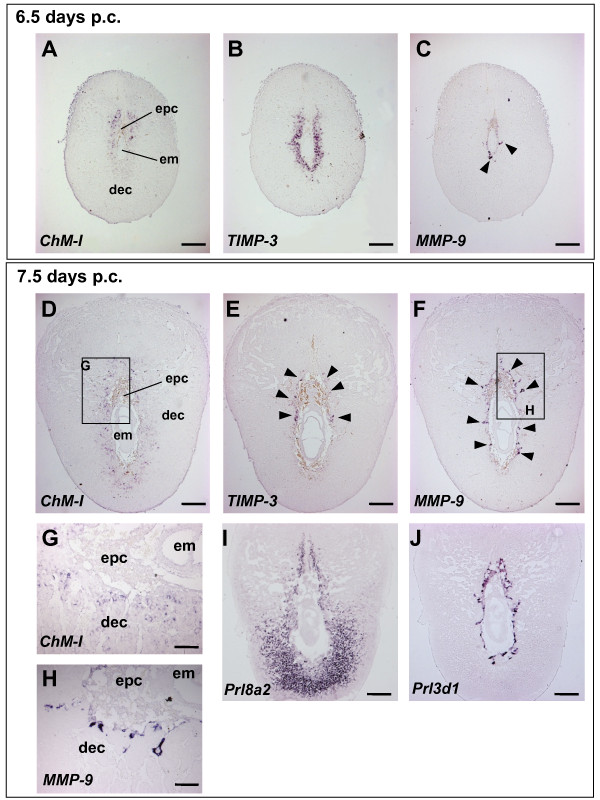
**Localization of ChM-I transcripts in the decidua**. Expression of *ChM-I *(A, D, and G), *TIMP-3 *(B and E), and *MMP-9 *(C, F, and H) transcripts analyzed by *in situ *hybridization using semi-serial sections of 6.5 (A-C) and 7.5 (D-F) days p.c. mouse decidual capsules. Expression of *Prl8a2 *(I) and *Prl3d1 *(a marker for trophoblasts; J) transcripts were also examined at 7.5 days p.c. Boxed areas in panels D and F are magnified in panels G and H, respectively. Note that, at 7.5 days p.c., *ChM-I *transcripts were broadly detectable in the primary decidua surrounding the implanting embryo and in *MMP-9*-positive cells (arrowheads in panel C and F), whilst *TIMP-3 *transcripts (arrowheads in panel E) were marginally detected at the mesometrial side. All images are representative of at least three independent experiments. *epc*, ectoplacental cone; *em*, embryo; *dec*, decidua. Bars in panels A-F and I-J, 300 μm; bars in panels G-H, 100 μm.

*In situ *hybridization revealed that the *ChM-I *transcripts were first detected around the EPC, which contains undifferentiated populations of trophoblast, at 6.5 days p.c. (Figure 2A). Interestingly we found that *TIMP-3 *was strongly expressed in the primary decidua, a mature portion of this structure, and that the localization of *TIMP-3 *transcripts was complementary to that of *ChM-I *(Figure 2B). At 7.5 days p.c., the site of *ChM-I *expression was considerably expanded to the entire primary decidua, whilst *TIMP-3 *expression was restricted to the thin layer of the primary decidua closer to the implanting embryo. The expression sites of these angiogenesis inhibitors were found to overlap with that of *Prl8a2 *(Figure 2I) and the invasive trophoblast giant cells that express a high level of *MMP-9 (matrix metalloproteinase-9) *and *Prl3d1 (prolactin family 3, superfamily d, member 1) *(Figures 2D-J) at the margin of the implantation chamber. By 8.5 days p.c., *ChM-I *transcripts were found to be expressed at a lower level around the EPC, where *MMP-9 *expressing trophoblast giant cells invade the decidua basalis to form the placenta (data not shown). These dynamic changes in the expression pattern of *ChM-I *are suggestive of their functional role in regulating the invasion of *MMP-9 *expressing trophoblasts.

### Localization of ChM-I protein in the decidua

**Figure 3:**
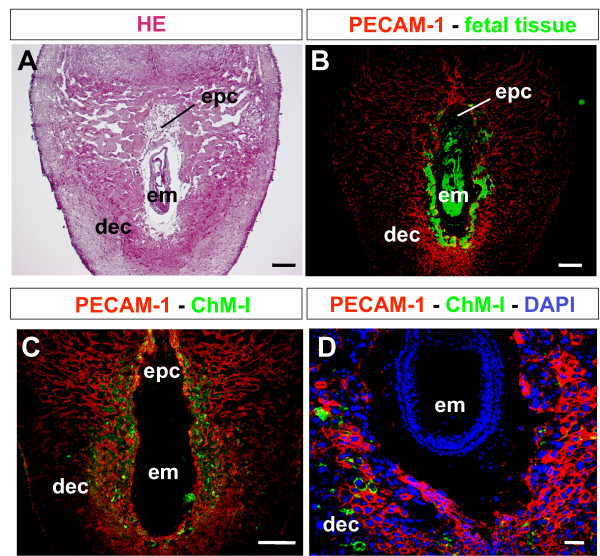
**Localization of ChM-I protein in the decidua**. (A and B) Visualization of invading fetal tissue through the decidua. Female mice were mated with homozygous EGFP transgenic male mice. At 7.5 days p.c., decidual capsules were dissected from the pregnant mice, fixed, and cryo-sectioned. Semi-serial sections were stained with HE (A) and anti-PECAM-1 antibodies (B, *red*), respectively. Fetal tissue (*green*) invading through the maternal decidua was observed under a fluorescence microscope. (C and D) Localization of ChM-I protein and PECAM-1-positive cells. Cryo-sections of 7.5 days p.c. tissues were double-immunostained with anti-ChM-I antibody (*green*) and anti-PECAM-1 antibody (*red*), and counterstained with DAPI (panel D). All images are representative of at least three independent experiments. *epc*, ectoplacental cone; *em*, embryo; *dec*, decidua. Bars in panels A-C, 200 μm; Bar in panel D, 50 μm.

Just after implantation, trophoblasts immediately invade the decidual matrix and form vascular connections with the maternal blood supply [12]. We therefore examined the localization of ChM-I protein in the decidua at 7.5 days p.c., comparing it with the distribution of maternal vascular networks and invading trophoblasts (Figure 3). To visualize the invading fetal tissue and the maternal vasculature, transgenic male mice ubiquitously expressing EGFP were mated with wild-type female mice, and the decidual sections were stained with anti-PECAM-1 antibody (Figures 3A and 3B). PECAM-1 immunoreactivity was detected in the whole decidua and was relatively strong in the primary decidua surrounding the EGFP-positive fetal invading cells (Figure 3B). Consistent with its mRNA localization (Figure 2), secreted ChM-I protein was detected in a punctate distribution in the primary decidua, where PECAM-1-positive maternal blood vessels and the invading trophoblasts may strongly interact with each other (Figures 3C and 3D).

### Expression of genes associated with angiogenesis and ECM remodeling in cultured decidual cells and EPCs

**Figure 4:**
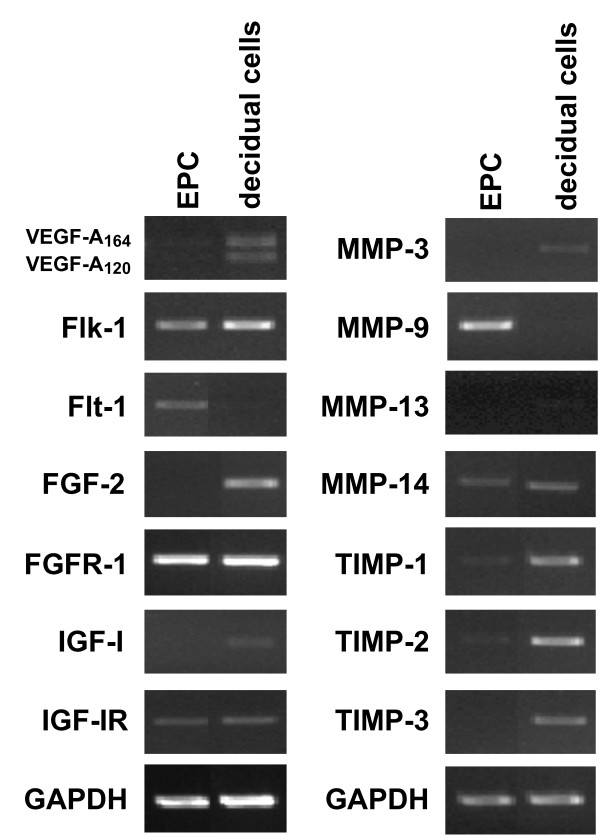
**Expression profiles of angiogenesis- and matrix remodeling-related genes in cultured EPCs and decidual cells**. EPCs and decidual cells were isolated from decidual capsules at 7.5 days p.c. Decidual cells (5 × 10^5 ^cells/well in 6-multiwell plates) and EPCs (10 EPCs/well in 6-multiwell plates) were cultured in DMEM containing 10% FBS for 2-5 days, respectively. Total RNA was isolated, reverse-transcribed, and the indicated sets of genes associated with angiogenesis (left panel) and tissue remodeling (right panel) were analyzed by RT-PCR (25 cycles for *VEGF-A, Flk-1, Flt-1, MMP-3, MMP-9, MMP-14, TIMP-1, TIMP-2, TIMP-3*; 30 cycles for *FGF-2, FGFR-1, IGF-I, IGF-IR, MMP-1*). *GAPDH *(25 cycles) was used as an internal control. The data are representative of three independent experiments.

Tissue remodeling and angiogenesis are two hallmark events that represent active tissue-tissue interactions at the feto-maternal interface during implantation and decidualization. Invading trophoblasts express MMPs to facilitate matrix remodeling, and respond to a number of growth factors produced by the decidua. Concurrently, the maternal vasculature is also stimulated and directed to the implanted embryo by angiogenic factors produced in the decidua [13-15]. RT-PCR indicated that decidual cells expressed angiogenic growth factors such as VEGF-A, FGF-2 (fibroblast growth factor-2), and IGF-I (insulin-like growth factor-I), whilst EPCs were positive for their cognate receptors (Figure 4). Moreover, EPCs predominantly expressed MMP-9, a molecule believed to be crucial for tissue remodeling. In contrast, cultured decidual cells expressed all of TIMPs that were examined (Figure 4). These results are compatible with the notion that trophoblasts are capable of responding to angiogenic and anti-angiogenic signal cues produced by the decidua.

### Induction of ChM-I expression by the decidualization of endometrial stromal cells *in vitro*

**Figure 5:**
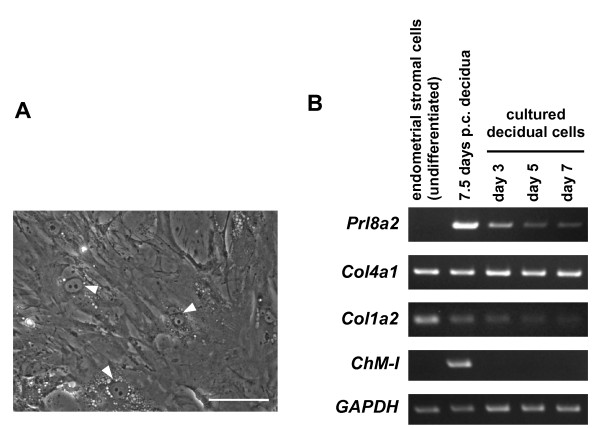
**Loss of ChM-I expression in cultured decidual cells**. (A) Decidual cells were enzymatically isolated from decidual capsules (7.5 days p.c.) and seeded at 5 × 10^5 ^cells/well in a 6-multiwell plate. The cells were then cultured for seven days in DMEM containing 10% FBS. Arrowheads indicate cells that have multiple- or enlarged nuclei, which are characteristic of the differentiated decidual cells. Bars, 100 μm. (B) Total RNA was isolated from decidual cell cultures at the indicated time points, endometrial stromal cells cultured for three days, and decidual tissue at 7.5 days p.c. One microgram of total RNA was reverse-transcribed, and the expression of *ChM-I, Prl8a2, Col4a1*, and *Col1a2 *was analyzed by RT-PCR (25 cycles for *Prl8a2, Col4a1, Col1a2*; 35 cycles for ChM-I). The data are representative of three independent experiments.

Decidualized endometrial stromal cells swell up their cell volume and produce basement membrane-like ECMs such as type IV collagen, laminin, and fibronectin instead of the interstitial matrices [16,17]. To investigate the expression of ChM-I in decidual cells, the cells were enzymatically isolated from the decidual capsules of mice at 7.5 days p.c., and cultured for seven days *in vitro *in the presence of 10% fetal bovine serum (FBS). As previously reported [18,19], the isolated cells were predominantly large, flattened, polygonal, and some were multi-nucleated (Figure 5A, arrowheads). The expression of ChM-I transcripts was monitored along with the detection of decidual cell-differentiation marker genes by RT-PCR (Figure 5B). While abundant type I collagen transcripts were detected only in undifferentiated endometrial stromal cells, a high level of type IV collagen was maintained in the cultured cells [17]. (Figure 5B). In contrast, the expression levels of *Prl8a2 *markedly declined (Figure 5B) during one week of culture, although no morphological changes of cells were apparent. Expression of *ChM-I *was readily detectable in the 7.5 days p.c. decidual capsules, but the capacity for *ChM-I *expression was only barely maintained when decidual cells were transferred into culture.

**Figure 6:**
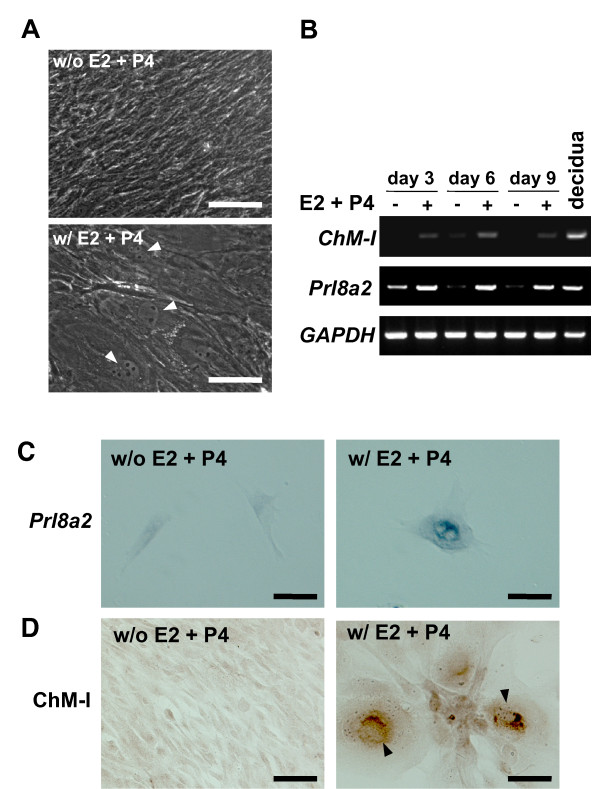
**Induction of ChM-I expression during the decidualization of mouse endometrial stromal cells *in vitro***. Mouse endometrial stromal cells were enzymatically isolated from the uteri of 4 week-old non-pregnant mice and cultured in medium containing 10% charcoal-stripped FBS. The *in vitro *decidualization of endometrial stromal cells was induced by the addition of E2 (0.1 nM) and P4 (100 nM) to the culture media. (A) Representative microphotographs of the endometrial stromal cells cultured for nine days in the presence (lower panel) or absence (upper panel) of E2 + P4. Arrowheads indicate enlarged or multi-nucleated cells that are characteristic of decidual cells. Bars, 50 μm. (B) Expression of *ChM-I *and *Prl8a2 *in decidualized cultures of endometrial stromal cells. Total RNA was extracted from the cells at the indicated time points and from mouse decidua (7.5 days p.c.). One microgram of each total RNA preparation was reverse-transcribed and analyzed by RT-PCR (35 cycles) for comparison. *GAPDH *was used as an internal control. (C, D) Expression of *Prl8a2 *gene in decidualized cells and immunostaining of ChM-I protein. The endometrial stromal cells were cultured for six days with or without E2 + P4, fixed, and subjected to *in situ *hybridization for *Prl8a2 *(C) and immunostaining with anti-ChM-I antibody (D). Note that intense signals for ChM-I protein (arrowheads) were observed in enlarged cells that also expressed *Prl8a2*. Bars, 30 μm. All images are representative of three independent experiments.

Kimura and coworkers have demonstrated previously that mouse endometrial stromal cells undergo decidualization *in vitro *in the presence of 17β-estradiol (E2) and progesterone (P4) [20]. We isolated endometrial stromal cells from 4-week-old immature female mice using their protocol [20], and cultured the cells with or without E2 and P4. Without E2 (0.1 nM) and P4 (100 nM), endometrial stromal cells retained the spindle-shaped morphology of fibroblasts throughout the culture period. However, when cultured in the presence of E2 and P4, these cells adopted rounded morphologies with enlarged and/or multiple nuclei (Figure 6A, arrowheads), resembling the decidual cells in culture shown in Figure 5A. RT-PCR analysis of total RNA from cells indicated that there was no apparent expression of *ChM-I *when the cells were cultured without E2 and P4 (Figure 6B). In contrast, *ChM-I *and *Prl8a2 *transcripts were induced by the addition of E2 and P4 to these cultures, even though the expression level was relatively low compared with that of the decidua at 7.5 days p.c. (Figure 6B). *In situ *hybridization to the cultured cells revealed that no hybridization signals were present in spindle-shaped endometrial stromal cells cultured without E2 and P4, but that hypertrophic rounded cells with enlarged and/or multiple nuclei formed in the presence of E2 and P4 exhibited positive signals for *Prl8a2 *mRNA as a result of decidualization (Figure 6C). Positive immunoreactivity to ChM-I protein was readily detected also in these decidualized hypertrophic cells (Figure 6D, arrowheads), but not without E2 and P4. These results indicate that ChM-I is induced in association with the decidualization of endometrial stromal cells.

### Effects of rhChM-I on the migration of Rcho-1 trophoblast cells

**Figure 7:**
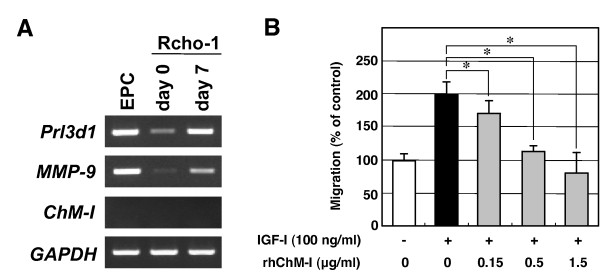
**Effects of rhChM-I on the IGF-I-induced migration of Rcho-1 trophoblast cells**. (A) Expression of *Prl3d1, MMP-9*, and *ChM-I *in Rcho-1 trophoblast cells. Rcho-1 trophoblast cells were seeded at 2 × 10^5 ^cells/well in 6-multiwell plates and cultured in growth medium (NCTC-135 medium containing 20% FBS) until reaching confluence (day 0; undifferentiated). The culture medium was then replaced with differentiation medium (NCTC-135 medium containing 10% horse serum), and the cells were cultured for seven days (day 7, differentiated) to promote their differentiation to trophoblast giant cells. EPCs were isolated at 7.5 days p.c. and cultured for two days in DMEM containing 10% FBS and 15 mM HEPES. Total RNA was then isolated and reverse-transcribed, and the expression of *Prl3d1 *(28 cycles), *MMP-9 *(28 cycles), and *ChM-I *(35 cycles) was analyzed by RT-PCR. *GAPDH *(28 cycles) was used as an internal control. The gel images are representative of three independent experiments. (B) Boyden chamber migration assay of Rcho-1 trophoblast cells. Serum-starved Rcho-1 trophoblast cells (1 × 10^5 ^cells/200 μl) were preincubated with or without rhChM-I for 20 min, and then seeded onto fibronectin-coated cell culture inserts in NCTC-135 medium containing 0.5% FBS. Chemotactic migration of Rcho-1 cells was induced by the addition of IGF-I (100 ng/ml) to the lower chamber. Cells were then allowed to migrate for 6 h in the presence of various concentrations of rhChM-I. Control cells were treated with 0.1% BSA/PBS and allowed to migrate in the absence of IGF-I. The number of cells that had invaded the undersurface of the insert was counted in five representative high power fields (under × 200 magnification) per insert. The values shown are percentages of the number of migrated cells compared with the control cells (22 ± 2.3 cells/field) and are the means ± SD of a triplicate assay. The data are representative of three independent experiments. The statistical significances were determined by the Student's t-tests as compared to IGF-I alone (*P < 0.05, one-tailed).

Taking advantage of rat Rcho-1 trophoblast cells, which are capable of recapitulating trophoblast giant cell differentiation *in vitro *[21], we examined whether recombinant human ChM-I (rhChM-I) affects the chemotactic migration of trophoblasts. In the growth medium, Rcho-1 trophoblast cells retained an undifferentiated state with a low level of expression of genes such as *Prl3d1*, a marker for trophoblast differentiation, and *MMP-9*, a terminal differentiation associated marker (day 0, Figure 7A). When the cells reached confluence, the culture medium was replaced with differentiation medium containing horse serum. Expression of these marker genes evidently increased by day 7 (Figure 7A). No *ChM-I *transcripts were detected in cells cultured in either growth or differentiation medium (Figure 7A). The effects of rhChM-I were then tested using modified Boyden chambers. Subconfluent Rcho-1 trophoblast cells cultured in growth medium were serum-starved and seeded onto migration inserts that had been coated with fibronectin, one of the abundant matrix components in the decidua. Among the angiogenic growth factors expressed in the decidual cells (Figure 4), FGF-2 (data not shown) and IGF-I, but not VEGF-A, exhibited a pronounced stimulatory effect on the chemotactic migration of Rcho-1 trophoblast cells. IGF-I induced the migration of these cells by approximately two-fold over the non-stimulated basal levels (Figure 7B). In contrast, the addition of rhChM-I to the cultures clearly inhibited the IGF-I-induced migration of the cells in a dose-dependent manner. At a dose of 0.5 μg/ml or higher, IGF-I-induced migration was completely abolished (Figure 7B).

### Anti-invasive effects of rhChM-I upon Rcho-1 trophoblast cells

**Figure 8:**
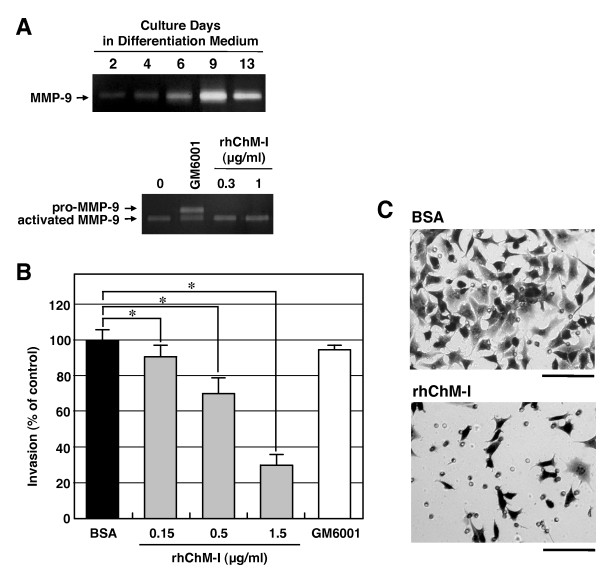
**Effects of rhChM-I on the invasion of Rcho-1 trophoblast cells**. (A) Effects of rhChM-I on the production and activation of MMP-9 in cultures of Rcho-1 trophoblast cells. Cells were grown to confluence, and the culture medium was then replaced with differentiation medium (NCTC-135 medium containing 10% horse serum). At the indicated time points (at day 2, 4, 6, 9, and 13), the culture medium was changed to medium containing 1% horse serum and conditioned for 24 h. The gelatinolytic activity in the conditioned medium was analyzed by gelatin zymography (upper panel). rhChM-I or GM6001 (25 μM) was added to cultures of Rcho-1 trophoblast cells in differentiation medium on day 9, and the cells were incubated another for 24 h in medium containing 1% horse serum. The gelatinolytic activity in the conditioned medium was then analyzed by gelatin zymography (lower panel). The gel images are representative of three independent experiments. (B) Matrigel invasion assay of Rcho-1 cells. Subconfluent Rcho-1 trophoblast cells were harvested and resuspended in NCTC-135 medium containing 0.5% horse serum, and seeded onto cell culture inserts (5 × 10^4 ^cells/200 μl) coated with a layer of Matrigel. The cells were allowed to invade for 16 h in the presence of 0.1% BSA/PBS (control), various concentrations of rhChM-I, or GM6001 (25 μM) in NCTC-135 medium containing 0.5% horse serum. The number of cells that had invaded the undersurface of the insert was counted under × 200 magnification. The values shown are percentages of the number of invading cells compared with the control cells (361 ± 20.7 cells/field) and are the means ± SD of a triplicate assay. The data are representative of three independent experiments with similar results. The statistical significances were determined by the Student's t-tests as compared to the control (0.1%BSA/PBS) (*P < 0.05, one-tailed). (C) Representative microphotographs of Rcho-1 trophoblast cells that had invaded the bottom surface of the insert in the presence of 0.1% BSA/PBS (upper panel, control) or 1.5 μg/ml rhChM-I (lower panel). Bars, 100 μm.

The invasive activity of Rcho-1 trophoblast cells was examined (Figure 8). Gelatin zymography revealed that Rcho-1 trophoblast cells produced the active form of MMP-9 in association with their differentiation to trophoblast giant cells when the cells are cultured in differentiation medium containing 10% horse serum (Figure 8A, upper panel). Activated MMP-9 was secreted as the predominant gelatinolytic activity, and MMP-2 and their zymogens were undetectable. GM6001 (25 μM), an inhibitor of broad spectrum MMP, partially reduced the activation of pro-MMP-9, but the addition of rhChM-I in culture produced no apparent effect on the formation of activated MMP-9 (Figure 8A, lower panel). Despite this, rhChM-I clearly inhibited the invasion of differentiated Rcho-1 trophoblast cells into Matrigel in a dose-dependent manner (Figure 8B). Rcho-1 trophoblast cells took on well-spread motile morphologies with active lamellipodia formation (Figure 8C, upper panel) and actively invaded a Matrigel layer in Boyden chamber (Figure 8B) under low serum conditions with 0.5% horse serum, which allow differentiation of the cells [21]. In the presence of rhChM-I, most cells took on quite distinctive static morphologies suggestive of their lower motility (Figure 8C, lower panel).

## Discussion

Along with the invasion of trophoblasts into the maternal endometrium, sequential decidual reactions occur and lead to the formation of two distinct zones: the differentiated zone proximal to an implanting embryo termed the primary decidual zone, and the distal peripheral zone consisting of immature mitotic decidual cells, termed the secondary decidual zone [22,23]. In our present study, we have demonstrated that ChM-I is expressed in the primary decidual zone during early pregnancy in mice. In contrast to *Prl8a2*, which is broadly expressed in decidual tissue and strongly induced during decidualization [10], *ChM-I *transcripts were localized to well-differentiated decidual cells in the primary decidual zone and were not detectable in the secondary decidual zone. Consistent with this, *ChM-I *was induced in cultured endometrial stromal cells that were undergoing decidualization in the presence of estrogen and progesterone *in vitro*. Both ChM-I protein and *Prl8a2 *mRNA were evident in the enlarged multi-nucleated well-differentiated cells in primary cultures of decidual cells [24].

The invasion of trophoblasts and maternal blood vessels are coordinately regulated in the primary decidual zone during early pregnancy. It has been known that the primary decidual zone remains to be vascularized prior to the invasion of trophoblasts [23]. Interestingly, *ChM-I *expression is predominantly detected at the mesometrial side of primary decidua facing the zone of vascularization, whereas the intense signals for *TIMP-3 *expression, another marker for well-differentiated decidual cells [25], were found at the antimesometrial side of primary decidua. When the trophoblasts were extensively detected along the margin of the implantation chamber at 7.5 days p.c., ChM-I was expressed at the entire zone of the primary decidua surrounding the invading trophoblasts, suggesting that ChM-I may participate in the invasion processes of trophoblasts.

The capacity for ECM degradation is one of the critical components required for the invasive behavior of cells [26,27]. Trophoblasts indeed express various kinds of proteinases, and proteinase inhibitors are localized in the decidua. For instance, it has been shown that TIMPs, cystatins, and plasminogen activator inhibitors are expressed in the distinct domains of the decidua [25,28,29]. In particular, MMP-9 and TIMP-3 are expressed at considerably high levels in the periphery of the implantation chamber and are thought to be pivotal regulators of ECM remodeling during the invasion of trophoblasts. However, trophoblast invasion proceeded relatively unhindered by the administration of a synthetic MMP inhibitor GM6001 into pregnant mice or the overexpression of TIMP-1 [14], while these treatments had led to a reduced size of the decidua and mesometrial displacement of an embryo. These observations are compatible with our present results showing that GM6001 had only a marginal inhibitory effect on the invasion of Rcho-1 trophoblast cells. TIMP-3 had also little impact on the Matrigel invasion of Rcho-1 trophoblast cells (data not shown). In contrast, ChM-I exhibited remarkable anti-invasive activity in the Matrigel invasion assay of Rcho-1 trophoblast cells, even though it did not interfere with the production and activation of gelatinases in the cells. The anti-invasive activity of rhChM-I was also evident in the Matrigel invasion of HUVECs [8], in which GM6001 was ineffective to block the invasion of cells (data not shown).

We have previously demonstrated that ChM-I inhibits the migration of vascular endothelial cells induced by angiogenic growth factors including VEGF-A, FGF-2, and IGF-I [8]. As suggested by the present RT-PCR study, EPCs are also capable of responding to these growth factors. Indeed, it has been reported also that IGF-I is a potent stimulator of mouse EPC cells and human trophoblast migration [13,30,31]. Similarly, IGF-I and FGF-2, but not VEGF-A (data not shown), stimulated the migration of Rcho-1 trophoblast cells in culture. Using a modified Boyden chamber assay, we found that rhChM-I completely abolishes the IGF-I-induced migration of Rcho-1 trophoblast cells. Morphologies of ChM-I-treated Rcho-1 trophoblast cells are also indicative of a critical reduction in cellular motility. This mode of ChM-I action is analogous to that on HUVECs [8]. Thus, ChM-I is likely to inhibit the invasion of Rcho-1 trophoblast cells and vascular endothelial cells by suppressing their cellular motility stimulated by IGF-I.

## Conclusions

The present study demonstrates that ChM-I is expressed in the primary decidual zone during decidualization at feto-maternal interface. Recombinant ChM-I inhibited the migration and invasion of Rcho-1 trophoblast cells in a manner independent of MMP activation *in vitro*.

## Methods

### Antibodies and reagents

A purified rat anti-mouse CD31 (PECAM-1) monoclonal antibody was purchased from BD Pharmingen (San Diego, CA). A rabbit anti-ChM-I polyclonal antibody was raised against recombinant human ChM-I expressed in CHO cells [32]. Alexa Fluor 594-conjugated goat anti-rat IgG and Alexa Fluor 488-conjugated goat anti-rabbit IgG antibodies were obtained from Molecular Probes (Eugene, OR). E2 and P4 were purchased from Sigma (St. Louis, MO). GM6001 (a broad-spectrum MMP inhibitor) was obtained from Merck KGaA (Darmstadt, Germany). Growth factor reduced BD Matrigel Matrix and mouse recombinant IGF-I was purchased from BD Biosciences (Bedford, MA) and R&D Systems (Minneapolis, MN), respectively.

### Expression and purification of recombinant human ChM-I

Recombinant human ChM-I (rhChM-I; corresponding to the region Glu^215^-Val^334 ^of the human ChM-I precursor) was expressed in a secreted form of an N-terminally FLAG-tagged fusion protein using the pCAGGS expression vector (generously donated by Dr. J. Miyazaki, Osaka University Graduate School of Medicine, Japan) [33]. The expression of rhChM-I under serum-free conditions and its purification from the culture supernatant were performed using the FreeStyle 293 Expression system (Invitrogen, Carlsbad, CA) and anti-FLAG M2 affinity gel (Sigma) according to the manufacturer's instructions [8].

### Tissue dissections

Pregnant and non-pregnant C57 BL/6 female mice were purchased from SHIMIZU Laboratory Supplies (Kyoto, Japan). All experimental procedures involving the use of animals for experimental purposes were approved by the Animal Care Committee of the Institute for Frontier Medical Sciences (Kyoto University) in accordance with institutional guidelines under the protocol number E-24-3. Mice were housed in a temperature and humidity controlled facility in Laboratory of Animal Experiments for Regeneration (Institute for Frontier Medical Sciences, Kyoto University) with a 12 h light/dark cycle and allowed access to food and water *ad libitum*. Female mice were checked for pregnancy by the presence of vaginal plugs in the morning (midnight = 0 day p.c.) and sacrificed by cervical dislocation. Uteri were removed from pregnant mice, cut open, and decidual capsules were then teased out. Using thin-needled forceps, embryos were removed from the decidua, and EPCs (ectoplacental cones; detectable as a cone-shaped vascularized region) were carefully separated from the distal endoderm at 7.5 days p.c. The placenta was separated from the C57 BL/6 pregnant mice (13.5 days p.c.). Some decidual samples or EPCs were prepared from the EGFP-transgenic female mice [34] (kindly provided by Dr. Masaru Okabe, Research Institute for Microbial Diseases, Osaka University, Japan) mated with wild-type C57 BL/6 male mice to track invading trophoblast cells as reported by Rosario et al [35].

### Cell culture

Isolation of decidual cells was carried out as described previously [36] with minor modifications. Briefly, decidual capsules were removed from ICR pregnant mice at 7.5 days p.c. and minced into small pieces in Hank's balanced salt solution-calcium/magnesium free. The minced tissues were then incubated at 37°C for 5 min in 0.05% type IV collagenase (Sigma) containing 0.02% deoxyribonuclease I (DNase I, Sigma), followed by incubation in 0.007% collagenase with 0.02% DNase I and 0.008% type XIV proteinase (Sigma) at 37°C for 5 min. The tissues were then triturated, and the supernatant was centrifuged at 1000 rpm for 5 min. Cell pellets were suspended in Dulbecco's modified Eagle medium (DMEM; Sigma) containing 10% FBS, and the cells were seeded at 5 × 10^5 ^cells/well in 6-multiwell plates.

Mouse endometrial stromal cells were isolated and cultured as previously described with minor modifications [20]. Briefly, uteri were removed from immature 4-week-old ICR mice, slit longitudinally to expose the endometrial surface, and minced into small pieces in PBS. The minced tissues were then incubated in 0.25% type IV collagenase (Sigma) and 0.3% bovine serum albumin (BSA; Sigma) in PBS for 1 h at 37°C. After enzymatic digestion, the supernatant was passed through a 40-μm cell strainer (BD Biosciences), centrifuged at 1000 rpm for 5 min, and the pelleted cells were rinsed with phenol red-free DMEM. The cells were then resuspended in DMEM supplemented with 10% charcoal-stripped FBS, 100 U/ml penicillin and 100 μg/ml streptomycin, seeded at 7.5 × 10^5 ^cells/well in 6-multiwell plates, and incubated in a humidified atmosphere of 5% CO_2 _in air for 1 h. After the 1 h incubation, unattached cells were gently removed with medium. For the *in vitro *decidualization, endometrial stromal cells were cultured with or without E2 and P4 at a concentration of 0.1 nM and 100 nM, respectively. The culture medium was changed every other day.

Rcho-1 trophoblast cells were obtained from Dr. M. J. Soares (University of Kansas Medical Center, Kansas City, USA) [21,37]. The cells were maintained in NCTC-135 medium (Sigma) supplemented with 10 mM HEPES, 50 μM β-mercaptoethanol (Sigma), 1 mM sodium pyruvate (Sigma), 50 μg/ml kanamycin sulfate (Sigma), and 20% FBS (growth medium). For the induction of differentiation, Rcho-1 cells in confluent culture were maintained in NCTC-135 medium containing 10% horse serum (GIBCO; differentiation medium). The culture medium was changed every other day. EPCs dissected from decidua at 7.5 days p.c. were cultured in DMEM supplemented with 10% FBS and 15 mM HEPES at 37°C under 5% CO_2 _in air.

### RNA preparation and RT-PCR

Total RNA extracts were prepared from tissues and cells using the single step method of Chomczynski and Sacchi [38]. First strand cDNA was synthesized from 1 μg of total RNA using SuperScript II RNase H- reverse transcriptase (Gibco BRL, Grand Island, NY). The amplification of glyceraldehyde-3-phosphate-dehydrogenase (GAPDH) gene was utilized as an internal control. The primers used for RT-PCR are listed in Table 1.

**Table 1:** Primers for RT-PCR analysis

**Genbank acc no**.	Gene	5'-Forward primer-3'	5'-Reverse primer-3'	Size (bp)
NM_010701	ChM-I	CATCGGGGCCTTCTACTTCT	CTGCGTCGTCCTGAACATTGGG	584

NM_010088	Prl8a2	TGAATGTCAAACAGGAGAGAA	CAATCTTGCCCAGTTATGCGG	628

NM_008864	Prl3d1	GCTTCCATCCATACTCCAG	TCGTTCTGAAAGACAACTCG	419

NM_009309	Brachyury	CTGCAGTCCCATGATAACTGGTCTAGC	CCAGGATTTCAAAGTCACATATATGTTGTAG	717

NM_009931	Col4a1	TGTCCAAGGCAACGAGCGTG	TGTTCTTCTCATGCACACTTGG	583

NM_007742	Col1a2	GAACGGTCCACGATTGCATG	GGCATGTTGCTAGGCACGAAG	401

NM_001025250	VEGF-A	CAGAGAGCAACATCACCATGC	TCCTCGAAGGATCTCCTCTTCC	313, 445

NM_010612	Flk-1	CACAGACACCACCGTGTACTCC	CTCCAAGGTAGACAGACTCGGC	516

NM_010228	Flt-1	TATGGTCTGTGTGCTTAGGTCG	ACAGGACCATCTATGGTCTTCC	476

NM_008006	FGF-2	AGCGGCATCACCTCGCTTCC	TGGAAGCAGTATGGCCTTCTGTCC	433

NM_010206	FGFR-1	AGAGACCAGCTGTGATGA	GGCCACTTTGGTCACACG	461

NM_010512	IGF-I	GGACCAGAGACCCTTTGCGGGG	GGCTGCTTTTGTAGGCTTCAGTGG	210

NM_010513	IGF-IR	ATGCTGTTTGAACTGCAGCGCATGTGCTGG	CCGCTCGAGCTTGCGGCCCCCGTTCAT	354

NM_010809	MMP-3	GAAATGCAGAAGTTCCTCGG	GAGTTCCATAGAGGGACTGA	878

NM_013599	MMP-9	TGTCATCCAGTTTGGTGTCG	TAGGGCAGAAGCCATACAGT	433

NM_008607	MMP-13	TGAAGAGACTGAGCGCTGCG	ATTCTTCCATGTGGTTCCAGCC	461

NM_008608	MMP-14	ATCATTGAGGTGGATGAGGAGG	CAGCCAACCAGGTACACTTGG	614

NM_001044384	TIMP-1	AGTAAGGCCTGTAGCTGTGCC	ACGAGGACCTGATCCGTCC	467

NM_011594	TIMP-2	AGCAGTGAGCGAGAAGGAGG	TCACTTCTCTCGATGCAGGC	450

NM_011595	TIMP-3	GCTACTGCAGCTGGTACCG	TTGGACTTCTGCCAACTTCC	518

NM008084	GAPDH	ACCACAGTCCATGCCATCAC	TCCACCACCCTGTTGCTGTA	452

### cDNA fragments for hybridization

A 739 bp fragment of mouse ChM-I (GenBank accession no. NM_010701) cDNA was amplified from cDNAs prepared from differentiated ATDC5 cells by RT-PCR and subcloned into pCRII TOPO TA cloning vector (Invitrogen). A 515 bp fragment of the mouse TIMP-3 (tissue inhibitor of metalloproteinase-3; GenBank accession no. NM_011595) cDNA and a 433 bp fragment of mouse MMP-9 (matrix metalloproteinase-9; GenBank accession no. NM_013599) were amplified from Mouse 17-day Embryo Marathon-Ready cDNA (Clontech, Palo Alto, CA). Both of the amplified cDNA fragments were then subcloned into the pCRII TOPO vector. Similarly, a 628 bp cDNA fragment of mouse Prl8a2 (prolactin family 8, superfamily a, member 2, also known as decidual/trophoblast prolactin-related protein; Gene accession number: NM_010088) was amplified using cDNA from 7.5 days p.c. decidua, and subcloned. A 551 bp cDNA fragment of mouse Prl3d1 (prolactin family 3, superfamily d, member 1, also known as placental lactogen-I; Gene accession number: NM_008864) was also amplified using cDNAs prepared from 7.5 days p.c. EPCs, and subcloned. The partial 624 bp cDNA fragment of mouse VEGF-A_164 _(Gene accession number: NM_001025250) was amplified from Mouse 11-day Embryo Marathon-Ready cDNA by RT-PCR and subcloned into pBluescriptII SK (+) at the NotI site. The primers used are listed in Table 2.

**Table 2:** Primers for cDNA fragment cloning

**Genbank acc no**.	Gene	5'-Forward primer-3'	5'-Reverse primer-3'	Size (bp)
NM_010701	ChM-I	GGGTCAATGGAAATAGATGCT	ACACCATGCCCAGGATGCGG	739

NM_011595	TIMP-3	GCTACTGCAGCTGGTACCG	TTGGACTTCTGCCAACTTCC	515

NM_013599	MMP-9	GATGCTATTGCTGAGATCCAGG	GGAAGATGTCGTGTGAGTTCC	433

NM_008864	Prl3d1	GACCTGTATACTCGTTTGGC	TCGTTCTGAAAGACAACTCG	551

NM_010088	Prl8a2	TGAATGTCAAACAGGAGAGAA	CAATCTTGCCCAGTTATGCGG	628

NM_001025250	VEGF-A	*GAAACCATGAACTTTCTGCTCTC	*GGTGAGAGGTCTGGTTCCCG	624

* The following sequence was added at the 5'-end of the primer. Added sequence: ATAAGAATGCGGCCGC

### Northern blot analysis

Total RNA (15 μg) was denatured with 6.5% formaldehyde, resolved on a 1% formaldehyde-agarose gel, transferred onto a nytran membrane (Schleicher & Schuell, Dassel, Germany), and cross-linked by UV irradiation. The cDNA fragments for hybridization probes were labeled with [α-^32^P] dCTP (Amersham Biosciences, Piscataway, NJ) by the random-primer method using a BcaBEST labeling kit (Takara, Shiga, Japan). Hybridization was carried out as described previously [39].

### *In situ *hybridization

Tissues samples were fixed with 4% paraformaldehyde (PFA)/PBS for 16 h at 4°C, embedded in paraffin, and cut at a thickness of 6 μm. The sections were dewaxed, rehydrated, treated with 10 μg/ml Proteinase K (Invitrogen) for 10-30 min at 25°C. For the cultured endometrial stromal cells, the cells were briefly fixed with 4% PFA/PBS at room temperature. Antisense and sense riboprobes for each gene were prepared with appropriate cDNA fragments, and labeled using a digoxygenin (DIG) RNA labeling kit (Roche, Mannheim, Germany). Hybridization was carried out at 50°C for 16 h in hybridization buffer containing 600 mM NaCl, 10% dextran sulfate, 1 × Denhardt's solution, 50% deionized formamide, 200 mg/ml tRNA, 10 mM Tris-HCl (pH7.5), and 1 mM EDTA. Sections were then rinsed under high stringency. Hybridization signals were detected immunohistochemically using an alkaline-phosphate-conjugated antibody, according to the manufacturer's instructions (Roche Diagnostics).

### Immunohistochemistry

Tissue samples were passed through a series of sucrose/PBS solutions (12%, 15%, and 18%), frozen in O.C.T. compound (Sakura Finetek & Tissue-Tek, Tokyo, Japan), and cryo-sectioned at a thickness of 8 μm. The frozen sections were then fixed with 4% PFA/PBS for 10 min at room temperature, rinsed with TBST (Tris-buffered saline containing 1% Tween 20), and incubated at 4°C overnight with primary antibody. For immunofluorescent staining, the sections were incubated with Alexa Fluor 488-conjugated goat anti-mouse IgG secondary antibody (1:300 dilution) or Alexa Fluor 594-conjugated goat anti-rat IgG secondary antibody (1:300 dilution), and 1 μg/ml of 4', 6-diamidino-2-phenylindole (DAPI, Sigma) in 2% skim milk/PBS. The slides were then washed with TBST and observed under a fluorescent microscope.

### Matrigel invasion assay

Both the upper and under surfaces of cell culture inserts (8 μm-sized pore filter; BD Biosciences) were coated with fibronectin (5 μg/ml; BD Biosciences) for 4 h at room temperature. An amount of 100 μl of 0.5 mg/ml growth factor-reduced Matrigel (BD Biosciences) in PBS was then added to the upper compartment of the insert, followed by an incubation for 6 h at room temperature to solidify the gel. When grown to subconfluence, Rcho-1 trophoblast cells were harvested with trypsin/EDTA, and re-suspended in NCTC-135 medium containing 0.5% horse serum at 2.5 × 10^5 ^cells/ml. The cells (5 × 10^4 ^cells/200 μl) were seeded onto the Matrigel-coated cell culture insert and incubated at 37°C in 5% CO_2 _for 16-18 h. The test reagent (rhChM-I or GM6001) was added to both the upper (200 μl) and lower chamber (600 μl). After incubation for 16 h, the cells were fixed for 15 min with 0.25% glutaraldehyde, and stained with Giemsa staining solution. Non-invading cells remaining on the upper surface of the insert were wiped off, and the number of cells that had invaded the undersurface of the insert was counted for five representative high power fields (under × 200 magnification) per insert.

### Cell migration assay

The migration of Rcho-1 trophoblast cells was evaluated using a modified Boyden chamber assay. The upper and under surfaces of the cell culture inserts (5 μm-sized pore filter, BD Biosciences) were coated with fibronectin (5 μg/ml) at 4°C overnight. Subconfluent Rcho-1 trophoblast cells were harvested and resuspended in NCTC-135 medium containing 0.5% FBS (1 × 10^5 ^cells/200 μl). The cells were then preincubated with or without rhChM-I for 20 min and seeded onto the upper surface of the inserts. Chemotactic migration of the cells was induced by the addition of IGF-I (100 ng/ml) in the lower chamber (600 μl). The cells were then allowed to migrate for 6 h at 37°C in the presence or absence of rhChM-I. The number of cells that had migrated to the undersurface of the insert was counted in five representative fields at a higher magnification (× 200 magnification) per insert.

### Gelatin zymography

Gelatinolytic activity in the conditioned medium of Rcho-1 trophoblast cell cultures was assessed by gelatin zymography. Rcho-1 trophoblast cells were seeded at 2 × 10^5 ^cells/well in 6-multiwell plates and grown to confluence in growth medium. The culture medium was then replaced with differentiation medium and the cells were maintained for a further 13 days. The cells were then conditioned at the indicated time points for 24 h in NCTC-135 medium containing 1% horse serum with or without test reagent. The conditioned media were centrifuged, and the supernatants were mixed with the same volume of × 2 non-reducing SDS sample buffer [125 mM Tris-HCl (pH6.8), 20% glycerol, and 4% SDS]. The samples were next separated on a 7.5% SDS-polyacrylamide gel containing 2 mg/ml gelatin (Sigma). After electrophoresis, the gel was rinsed twice in 2.5% Triton X-100 to remove the SDS, then incubated in 50 mM Tris-HCl (pH8.0) buffer containing 5 mM CaCl_2 _to allow gelatinolysis for 16-20 h. Gels were stained with Coomassie Brilliant Blue R-250. The clearing of background gelatin by proteinases revealed gelatinolysis as clear bands.

## Authors' contributions

SM participated in the design of the study, performed the main experimental work, analyzed the data, and drafted the manuscript. CS conceived the study, participated in its design coordination and carried out northern blot analysis. KM and JK carried out the cell invasion assay and analyzed the data. YH coordinated the study and wrote the manuscript. All authors read and approved the final manuscript.
